# Marfan syndrome with multiseptate pneumothorax and mandibular fibrous dysplasia

**DOI:** 10.4103/0970-2113.56353

**Published:** 2009

**Authors:** A. Kate, D. Gothi, J. M. Joshi

**Affiliations:** *Department of Respiratory Medicine, T. N. Medical College, B. Y. L. Nair Hospital, Mumbai, India*

**Keywords:** Marfan syndrome, firous dysplasia, pneumothorax

## Abstract

We describe a rare case of pneumothorax due to Marfan syndrome associated with fibrous dysplasia of the mandible. Marfan syndrome and fibrous dysplasia were possibly due to a common etiological factor. The association between the two and other tumors described in literature related to Marfan syndrome is discussed.

## INTRODUCTION

Marfan syndrome is an autosomal dominant heritable disorder of connective tissue. Pneumothorax is a common pulmonary complication of Marfan syndrome. Marfan syndrome is known to be associated with various tumors, like collagenous fibroma of the palate, odontogenic keratocyst, and fibromuscular dysplasia. We describe a case of Marfan syndrome with fibrous dysplasia of the mandible, possibly due to a common etiological factor.

## CASE REPORT

A 32-year-old man was referred from a dental hospital for preoperative pulmonary evaluation of recent-onset dyspnea on exertion. His chest radiograph showed right-sided pneumothorax. He was undergoing treatment from the dental hospital for the right mandibular swelling and recurrent dental infections. He also had reduced vision in both the eyes. Intraocular lens implantation was performed for him in the right eye for ectopia lentis at the age of 13 years.

**Figure 1:**
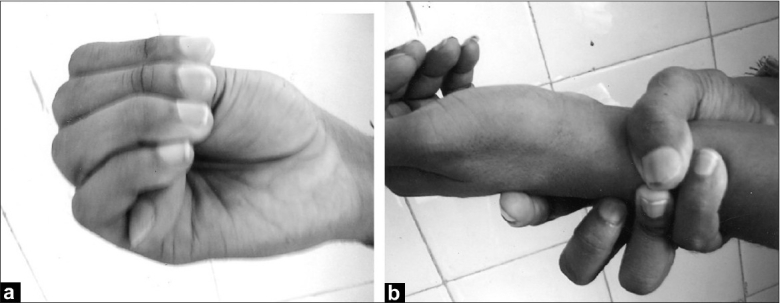
(a) Steinberg sign (thumb sign); (b) Walker sign (wrist sign)

Physical examination revealed reduced upper-to-lower body segment ratio, which was 0.71 (normal, 0.86); positive Walker [Figure 1a] and Steinberg signs [Figure 1b]; and a high arch palate. Respiratory system examination showed reduced movement, a hyper-resonant note, and reduced breath sounds over the right hemithorax. On ophthalmology evaluation, the vision in the left eye was limited to perception of light, whereas that in the right eye was 6/60; and posterior chamber intraocular lens was detected in the right eye. B-scan ocular ultrasonography showed retinal detachment in the left eye.

**Figure 2:**
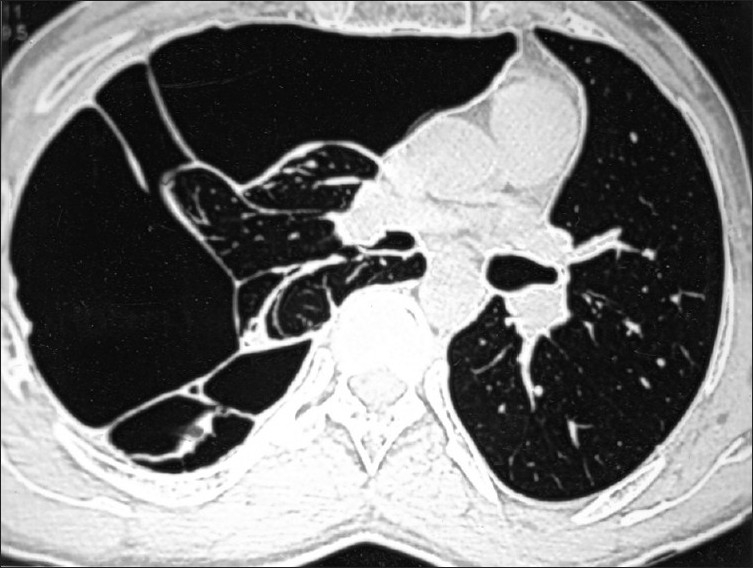
High-resolution computed tomography of thorax showing loculated multiseptate pneumothorax on the right side

**Figure 3:**
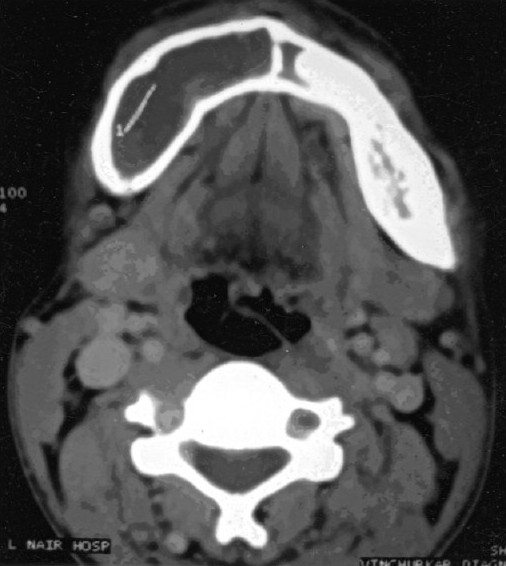
Computed tomogram of mandible showing large expansile lytic lesion in the right hemimandibular region

**Figure 4:**
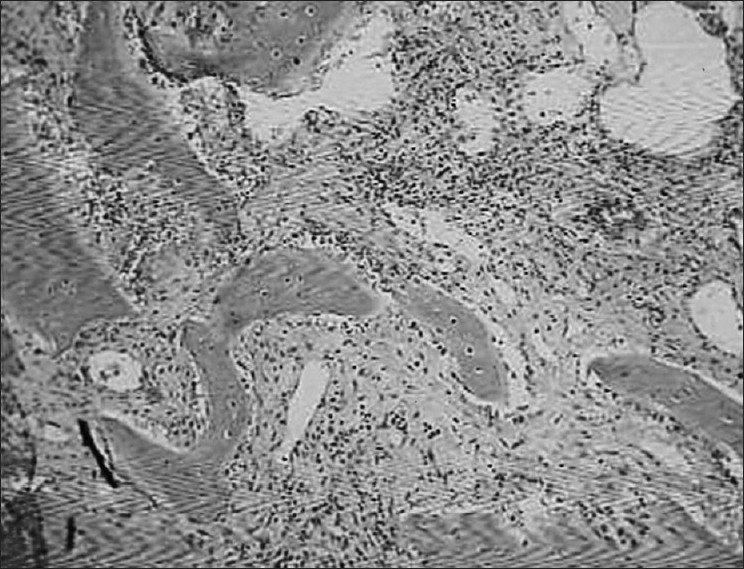
Incisional biopsy of mandible showing fibrous dysplasia

His hematological investigations showed hemoglobin of 13.5 gm%, total leukocyte count of 8,200/μL, serum calcium of 8.6 mg/dL (normal, 9.2-11 mg/dL), serum phosphorus of 2.8 mg/dL (normal, 2.3-4.7 mg/dL), and serum parathyroid hormone of 42.78 pg/mL (normal, 10-65 pg/mL). Radiograph of both hands showed periarticular osteopenia with metacarpal index of 8.79 (normal, <8.4). Two-dimensional echocardiogram revealed mild mitral valve prolapse. High-resolution computed tomogram thorax was suggestive of loculated multiseptate pneumothorax on the right side [Figure 2], and the lung fields were normal on the other side. Computed tomogram of mandible [Figure 3] showed large expansile lytic lesion in the right hemimandibular region with a smaller lesion in the left hemimandible. Incision biopsy of the lytic lesion was suggestive of multiple noncommunicating bony trabeculae, which were variably shaped (“C”, “Y”, and “H”) and lined by osteoblasts [Figure 4]. The intertrabecular stroma was loose and at places fibrous with dense focal collection of plasma cells, suggestive of fibrous dysplasia of mandible. He was treated with intercostal tube drainage for the multiseptate pneumothorax; the lung, however, did not expand completely. The patient was referred back to the dental hospital for resection of the mandibular fibrous dysplasia after he improved symptomatically.

## DISCUSSION

Marfan syndrome is a connective tissue disorder with clinical variability and pleiotropic manifestations. The diagnosis is based on Ghent diagnostic criteria.[1] In adults, the combination of the major criteria in two different body systems and minor criteria in the third system amongst the cardiovascular, skeletal, ocular, pulmonary, skin, nervous systems provides the clinical diagnosis in majority of the cases. In children, genotyping may contribute to the diagnosis, especially if family history is negative. The clinical manifestations are due to mutation in the fibrillin-1 (FBN-1) gene located on chromosome 15Q21.[2] It is inherited in approximately 75% of cases and occurs due to spontaneous mutation in the remaining 25%.[3] More than 150 different mutations of the FBN-1gene have been isolated, and it may be that each family has a unique genetic mutation for the syndrome.[4] This could explain the considerable variability in the clinical presentations of Marfan syndrome. It also means that diagnosis cannot necessarily be made from genetic testing alone. In our case, diagnosis of Marfan syndrome was based on two major criteria and one minor skeletal criterion; one major criterion and two minor ocular criteria; and one minor cardiovascular and pulmonary criterion each.

Fibrous dysplasia of bone is a developmental anomaly in which normal bone is replaced with fibrous connective tissue. As the lesion matures, the fibrous connective tissue is replaced with irregularly patterned trabecular bone. The abnormality is of bone-forming mesenchyme that manifests as a defect in osteoblastic differentiation and maturation. Virtually any bone in the body can be affected. Four disease patterns are recognized: Mono-ostotic form (74%), polyostotic form (13%), craniofacial form (13%), and cherubism. The lesions of fibrous dysplasia are twice as common in the maxilla as the mandible. Three radiological presentations of mono-ostotic fibrous dysplasia have been described: 1) the pagetoid or ground-glass pattern (56%) of bone; 2) the sclerotic pattern (23%), which is a uniformly dense bony change; and 3) the cyst-like pattern (21%). These bone cavities are analogous to simple bone cysts.[5] Fibrous dysplasia is generally self-limiting and does not require treatment except for cosmetic reasons, infection, pain, discomfort, fractures, and nerve entrapment.[6] If treatment consisting of recontouring or resection is undertaken, it should be postponed until after cessation of skeletal growth, since early treatment may accelerate growth of the lesion.[7]

Marfan syndrome has been described with various tumors in literature, like collagenous fibroma of the palate,[8] odontogenic keratocyst, and fibromuscular dysplasia.[9] However, its association with fibrous dysplasia has not been reported. The simultaneous presence of two rare diseases could be either coincidental due to a spontaneous mutation or caused by a common etiological factor. An association of Ehlers-Danlos syndrome (EDS) and mono-ostotic fibrous dysplasia[10] has been described. Since EDS and Marfan syndrome are the disorders of connective tissue and bony dysplasia has been described in EDS, the fibrous dysplasia of mandible in our case could be due to a common etiological factor.
